# Long‐term consumer involvement in cancer research: Working towards partnership

**DOI:** 10.1111/hex.13258

**Published:** 2021-05-05

**Authors:** Kristi Milley, Sophie Chima, Jennifer G. McIntosh, Elle Ackland, Jon D. Emery

**Affiliations:** ^1^ Department of General Practice Faculty of Medicine, Dentistry and Health Sciences Primary Care Collaborative Cancer Clinical Trials Group Centre for Cancer Research University of Melbourne Victorian Comprehensive Cancer Centre Melbourne Vic. Australia; ^2^ Department of General Practice Faculty of Medicine, Dentistry and Health Sciences Centre for Cancer Research University of Melbourne Victorian Comprehensive Cancer Centre Melbourne Vic. Australia; ^3^ Primary Care Collaborative Cancer Clinical Trials Group Community Advisory Group Melbourne Vic. Australia

**Keywords:** cancer research, patient and public involvement, qualitative interviews

## Abstract

**Background:**

Meaningful consumer involvement in health research is important. There are limited data on how to maintain long‐term consumer involvement.

**Objective:**

To identify barriers and facilitators to meaningful long‐term consumer involvement in research.

**Design:**

Six semi‐structured interviews were conducted with members of the Primary Care Collaborative Cancer Clinical Trials Group (PC4) Community Advisory Group (CAG) and included the review of 40 supporting documents. Interviews and documents were analysed using inductive thematic analysis; the themes were mapped onto the domains of Cancer Australia's National Framework for Consumer Involvement in Cancer Control.

**Results:**

Equality, respect and feeling valued were facilitators to long‐term involvement. These elements were part of an overarching theme of organizational commitment. Creating balance, managing competing deadlines and integrating a consumer role with a personal life were key barriers to involvement. These themes mapped strongly to the National Framework for Consumer Involvement in Cancer Control domains of committed organizations, capable consumers, inclusive groups and shared focus.

**Conclusion:**

Research networks should reflect on several factors to maintain long‐term consumer involvement. Networks should aim to build a meaningful relationship, using clear communication and education, that reinforces the value and scope of a consumers contributions. We found that consumer education needs do not diminish over time and adequate skill development, support and feedback need to be on‐going. Creating regular opportunities for feedback and reflection are important to continue to meet best practice guidelines.

## 1 INTRODUCTION

Consumer involvement is an essential element of high‐quality research. In the UK, the idea of including patient voices in health policy and research was first suggested the 1970s.^1^ Over the past 15 years, the importance and evidence of meaningful involvement with consumers have continued to evolve and grow.^2^, ^3^ It has become central to the research policy agenda in Australia, the United Kingdom, Canada and the United States of America.^4^, ^5^ Core to this partnership is the concept that patients, carers or family and friends affected are research collaborators not passive participants. This partnership is an important mechanism for increasing public confidence in cancer research.^6^, ^7^

The benefits of involving patients and the public in the development and conduct of research include improved quality, appropriateness of study design, improved ethical acceptance, improved implementation and dissemination of results^8^, ^9^, ^10^, ^11^, ^12^, ^13^ as well as increased accountability for publicly funded research.^14^

In cancer research, involvement is focused on improving outcomes for patients by developing studies that better align with patients’ needs, increasing acceptability and reducing the burden for patients participating in research studies.

## 2 CONTEXT

In Australian cancer services and health and medical research, ‘consumer’ is the most common nomenclature when describing any person affected with or by cancer. This may be a patient, survivor, carer or family member. In this context, consumer and consumer involvement are synonyms for Patient and Public Involvement (PPI), the public, service users and end users.

Cancer Australia is an Australian Federal Government agency that works to reduce the impact of cancer on all Australians and supports research and clinical trials [https://www.canceraustralia.gov.au/]. This includes funding 13 multisite collaborative cancer clinical trials groups, including the PC4 [http://pc4tg.com.au/]. PC4 is a national organization that supports the development of new clinical trials focused on the role primary care plays across the cancer continuum. This support includes the facilitation of consumer involvement in the development of research priorities, new research concepts and grant funding applications.

In 2011, Cancer Australia produced the National Framework for Consumer Involvement in Cancer Control.^15^ The framework was developed to improve the ‘meaningful consumer involvement at all levels of cancer control in order to improve outcomes and experiences for people affected by cancer engagement’. The word meaningful is an important distinction that acknowledges that consumers’ input is integral to the research development process and that their involvement must extend beyond being superficial or tokenistic. The framework identifies keys elements that are designed to help organizations more effectively engage with consumers. Consumers can participate in research at five different levels, informing, consulting, involving, partnership and consumer‐led research.

There is a paucity of research that investigates the evidence related to consumer involvement activities including long‐term involvement of consumers.^16^, ^17^ Much of the current evidence investigates the initiation of consumer involvement or the organizational approach to beginning consumer involvement.^3^ Given the benefits of consumer involvement^8^, ^9^, ^10^, ^11^, ^12^, ^13^ in the development of health and medical research, the purpose of this study was to identify barriers and facilitators to long‐term consumer involvement within a well‐established consumer involvement framework in the context of cancer in primary care research.

## 3 METHODS

PC4 established a joint Community Advisory Group (CAG) in 2010. This group of consumers was shared with the Psycho‐oncology Co‐operative Oncology Group (PoCoG) another cancer clinical trials group funded by Cancer Australia. Eligible participants were current or recently resigned members (within the last 6 months) of the CAG. All eleven eligible members were invited to participate in the study via email. Members who consented were interviewed over the phone using a semi‐structured style. The question guide was produced through an iterative process (KM, SC & JM) and was informed by the operational documents related to the CAG.

### 3.1 Data collection

Semi‐structured, one‐on‐one, telephone interviews were conducted by a female research assistant external to PC4 with significant previous experience and training in conducting qualitative interviews. Participants’ demographics including length of time they served on CAG, were collected. Interviews took place between December 2017 and July 2018. All interviews were conducted using Redback Teleconferencing and were recorded with the participant's permission. Interviews were professionally transcribed verbatim and anonymized (Pacific Transcriptions). All documents related to the CAG were reviewed including minutes from CAG meetings, workshops, teleconferences, internal and external communications containing direct feedback and experiences expressed by CAG members. Reasons for member resignation were also identified within personal emails and meeting minutes. Additionally, the past CAG Chair and an additional original CAG member were consulted to support information extracted from documents in particular the reason for past CAG members’ resignations. The datasets generated during and/or analysed during the current study are available from the corresponding author on reasonable request.

### 3.2 Topic guide

Interviews explored domains related to (i) participants’ prior experience in volunteering, (ii) their motivations for joining the PC4 CAG, (iii) their motivation to continue their consumer role and (iv) personal and organizational barriers and facilitators to their continued involvement.

### 3.3 Ethical **review**

The project was granted ethical approval from the University of Melbourne Human Ethics Sub‐Committee (ID 1750496.1).

### 3.4 Data analysis

This study used a hybrid approach to analysis.^18^ Inductive coding was first applied to the raw data to generate codes and themes. This approach involved the stages of familiarization with the data, generating initial codes, searching for themes, reviewing themes, defining and naming themes and producing a summary.^19^ A deductive approach was then used with to map these themes to the domains and subdomains of Cancer Australia's National Framework for Consumer Involvement in Cancer Control^15^ as an a priori template and to evaluate their responses against a comprehensive standard. Two researches (KM & SC) used an inductive approach to thematically analyse interviews.^19^

To ensure reliability, a process of inter‐coder consensus was adopted. Each coder independently coded 3 transcripts and developed a coding frame. Codes within this coding frame were reviewed before all remaining transcripts were coded. Final themes were revised and agreed through an iterative process. Coding and themes were organized using NVivo software (QSR International, V11). To supplement this, a similar process was used to review supporting documentation. For example, meeting minutes were reviewed similarly to a transcript with any reference to personal statements made by CAG members highlighted and coded.

Following this, themes identified were mapped to the National Framework for Consumer Involvement in Cancer Control. This framework was developed in 2011 in collaboration with Cancer Voices Australia.^15^ This framework has four interconnected domains that together are the foundation for sustainable and meaningful consumer involvement. These domains are committed organizations, capable consumers, inclusive groups and shared focus.

## 4 RESULTS

Between 2010 and 2018, there were a total of 18 CAG members, with an average length of service of four years (range 2 months‐8 years). Eleven members were eligible to be contacted, that is a current member or a member who had resigned in the past six months. The remaining eight members had resigned at least two years prior to the study period. Seven of the 11 members consented and six were available for interview. Of the four members that declined participation no reason was provided by two members, one member was on a Leave of Absence at the time of the study, and the final member was too busy to participate. All four members were well‐established CAG members with their length of service ranging from 3.5 years‐7.5 years. The interviews lasted between 27 and 73 minutes. The total amount of audio data was four and a half hours. Forty text documents were also reviewed, these included drafts of CAG terms of reference, peer support programme documentation and evaluation, consumer email communications and meeting minutes, between 2010 and 2018. Since 2011, nine CAG members resigned. Of these, one member took on a leave of absence and was not heard from again, one member resigned due to a perceived conflict as a consumer following completion of a PhD, one member was asked to leave due to a breach in the Code of Conduct. The remaining six members resigned due to increased commitments with family or work.

All participants revealed previous volunteering experience prior to joining the CAG.

**FIGURE 1:**
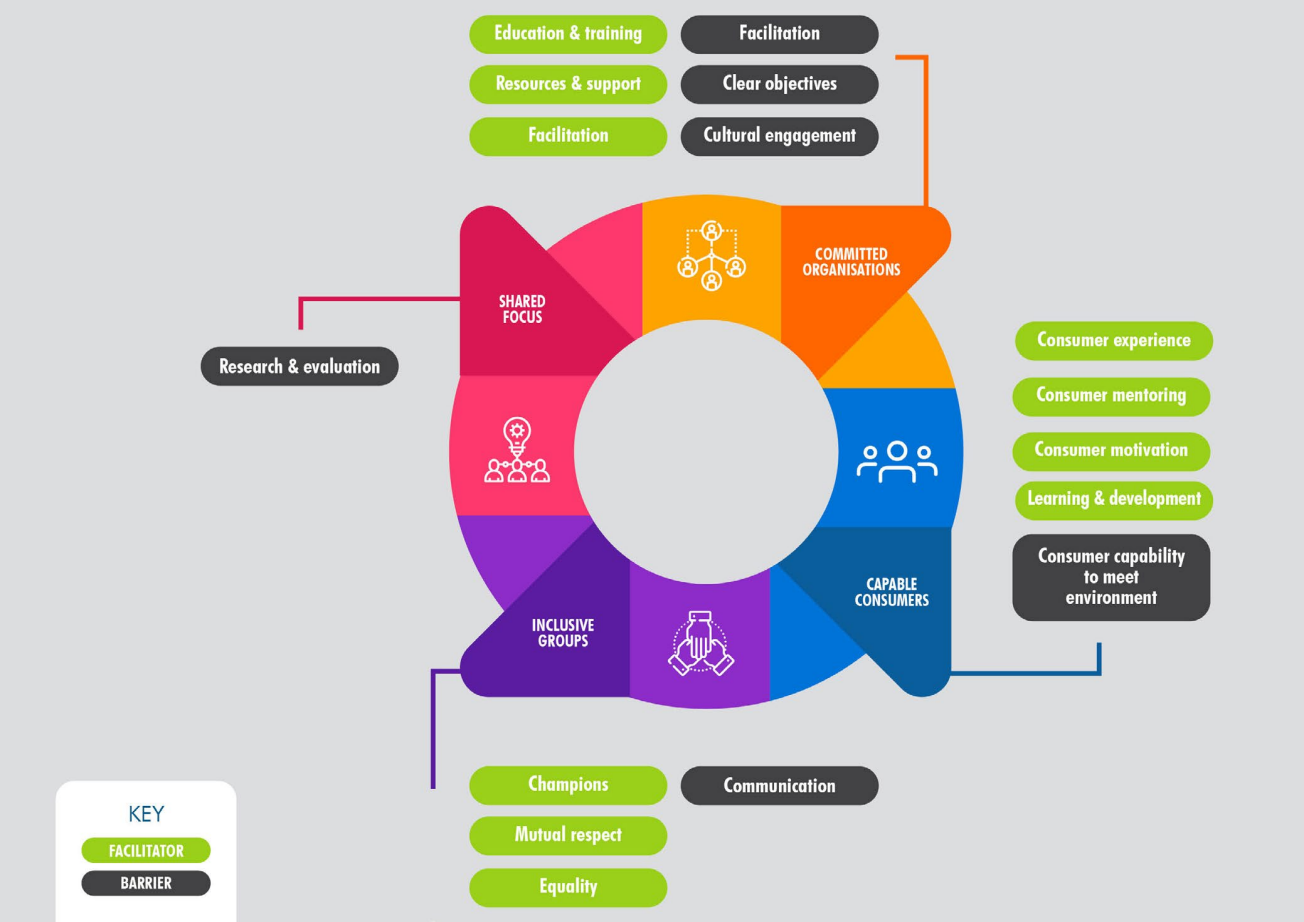
Facilitators and barriers identified to long‐term consumer engagement based on the Cancer Australia framework^15^

Based on Cancer Australia's framework, themes identified mapped most strongly to committed organizations, capable consumers and inclusive groups. While the domain of shared focus did not map strongly to consumer experiences and discussions (Figure 1).

### 4.1 Committed organizations

This domain contains seven subdomains, three of which, resources and support, education and training, and facilitation, were discussed by consumers.^15^ They acknowledged investment from PC4 as a facilitator to their continued involvement. This investment included providing resources and support, education and training and facilitation. ’There's a significant contribution, they fund my travel, my accommodation and time to be there… and there's been elements of training. So, they continually check with us, do you need more?’ (C2). Members also highlighted that active facilitation in coordination of consumer activities was a facilitator ’I'm impressed really with the way they do workshops, the way they structure meetings’. (C2). Facilitation was also described as a barrier in particular a lack of involvement in participation in the development of organizational strategy ‘we've not ever been asked about what we see as gaps’ (C3). Consumers also raised a lack of clear objectives and cultural involvement as organizational barriers to involvement ‘diversity, outcome driven ‐ which I don't think has been particularly good up until now’ (C6).

### 4.2 Capable consumers

Four of the eight subdomains featured as facilitators to involvement: consumer experience, motivation, learning and development, and mentoring.^15^ Consumers expressed that their unique experience was where their value lay, and it was something ‘that can't come from anywhere else’ (C3). They expressed that this made them continue wanting to engage with new research. Many outlined that their motivation to join stemmed from an altruistic desire to improve outcomes and experiences of new cancer patients. This motivation was sustained by a desire to be a voice for those that may be underrepresented. Their experiences in engaging with other cancer support communities contributed to their motivation ‘I meet people who they don't have very good English or low socioeconomic status… I think it's really incumbent upon us who are able to do it to be that voice’ (C1).

A mentor or buddy was also a facilitator to feeling more capable as a consumer and being prepared to engage with research, ‘I think oh I'm not sure about this one, so usually I ring my friend and have a consult with her’ (C2).

A barrier to feeling capable was difficulty in engaging in research based on a different lived experience or lack of understanding of the research. This was particularly important when research was about a cancer type different to their lived experience ‘…certainly, in some of the other tumour streams you think well, I just don't know enough about this’ (C1). It was suggested that this led consumers to feel that they could or should not contribute.

### 4.3 Inclusive groups

The feeling of inclusion and respect was strongly iterated by many consumers and permeated their sentiments across domains.^15^ They felt that researchers were always ‘…willing to listen…, to take it on board and show a lot of respect’ (C4) and that the organization encouraged champions who promoted consumer involvement to other researchers. Importantly, they also expressed they felt like equal contributing members in the development of research ‘PC4 do that very well…liaise …with the researchers and with us and make sure they're constantly in ‐ that everybody feels part of that whole’ (C3). In contrast, the quality of information provided was a barrier to their involvement, ‘…sometimes the quality of the information we get sometimes is not as good as it should be in order to make good decisions and informed reviews’ (C6).

### 4.4 Shared focus

This domain did not feature prominently in experiences of consumers.^15^ The only area described by consumers was that in some cases, research and evaluation felt like a barrier. They did not feel they understood the organizations research priorities and how these were developed. Some consumers felt that ‘…research projects come up on the whim of a researcher’ and that ‘…there's a lack of cohesiveness of direction in research itself’ (C2).

Permeating throughout the experiences described by consumers was a feeling their continued involvement with the organization was due to them feeling valued and that their contributions felt like they were meaningful. Meaningful in the sense that what they said made an impact on the design and conduct of research and that research improved outcomes for cancer patients. Consumers felt they were able to see the impact of their own involvement. This sentiment very strongly aligned with why many described their motivation for initially volunteering, to improve the experience for the next cancer patient, to share their experiences so that other people may have better outcomes – ‘…they keep telling the consumers how valuable we are to them and how our voice makes a difference to their research. So, I see it's no longer tokenism, it's actually realistic’ (C2).

Lastly, a review of our organizational documentation revealed that the only reason consumers left the organization was due being unable to manage a balance between their CAG commitments and their personal life. This was reinforced by current members who frequently emphasized the importance of balancing and the timing of their workload ‘I think there's a big gap between when things come through, and sometimes you can be given three or four things at once’ (C6). They also emphasized the importance of having realistic turn‐around times when providing input or feedback into research. ‘A couple of times I feel bad because I haven't been able to contribute to the deadlines for doing things. If I've had other things on’ (C3). This was often expressed with an understanding of the short time frames that are imposed on researchers themselves.

## 5 DISCUSSION

It is clear from our results that the concept of meaningful involvement is important to consumers and is a key facilitator to their long‐term involvement in cancer in primary care research. Elements described by consumers which underpin this concept included organizational commitment, visible champions, equality, respect, inclusive groups, support to enable capable consumers and understanding workload. Consumers felt valued, identifying that their personal and unique experience contributed something important to research and could see their input in the research outcomes. They felt supported by the organization both by the team and their peers but also financially. Despite this, some reported they were not given the opportunity to provide feedback about the research strategies which sometimes seemed to be arbitrary and to ‘come from nowhere’. They also commented there was a lack of diversity represented in the research that should be addressed.

In our study, nearly two‐thirds of eligible CAG members consented to the study. Our results only reflect the experiences of this subset of our CAG. This limitation means that it is possible that those that participated were more positive and engaged. Two members did not participate due to other commitments which could indicate a lack of engagement. Future research could aim to engage more members to ensure barriers and facilitators are more broadly representative. A strength of this study is the length of involvement of CAG members. Our results reflect involvement over a period of up to eight years.

A strategic review of health and medical research in Australia identified that consumer involvement needs to be underpinned by strong leadership to ensure meaningful involvement.^20^ The outcomes of meaningful involvement include shaping research topics that improve outcomes important to consumers.^21^ In this study, leadership was discussed by consumers in relation to both organizational commitment and visible champions. The results were positive about the level of leadership and how, from a consumer perspective, it was supportive of long‐term involvement.

Organizational commitment has been highlighted as a driver of involvement by other cancer research networks.^22^ Similar to our findings, part of this commitment included support that provided out‐of‐pocket expenses, travel and education.^23^ These facilitators have been described broadly in terms of involvement and our results suggest these facilitators are important for sustained involvement.

The Cancer Australia Framework also suggests that consumers can be involved in research at five different levels with the lowest level being information moving up through the pyramid to consulting, involving, partnership, topped by consumer‐led participation. These levels are related to both consumer capability and organization capacity. The themes identified in this study suggest that as an organization, PC4, has achieved ‘involvement’ of consumers but has yet to move past this towards ‘partnership’ or ‘consumer‐led’ research. This is suggested by the results within shared focus where consumers did not feel they were part of the development of PC4’s research priorities and that research is developed on the whim of a researcher. There is clearly room for development within our existing consumer involvement model to create more versatility and responsiveness to ensure better consumer representation in the development of research priorities but also at different stages of research project development.

Consumers referenced champions, equality and respect as important for creating supportive research development processes which ultimately empower consumers in their role. There was no reference to any tension between themselves and/or the organization which further suggests the environment was a supportive one. This is particularly important as tension has been highlighted in other settings as a barrier to consumer involvement,^24^ and we would argue to sustained involvement as well.

The resource and time investment in developing structures and processes for authentic consumer involvement is intensive.^3^, ^4^, ^25^ These results highlight that this investment is on‐going and that periodic reflection of the organizational approach to consumer involvement may be helpful to identify if current approaches continue to meet needs or if a new or updated structure should be implemented. The reflection undertaken by our organization following this study highlights this. In response, to this study PC4 created a Community Network. This network allows members to select what activities they would like to participate in. The network was designed to reduce workload for CAG members but to work towards increasing consumer diversity. This study highlights that long‐term involvement of consumers is a return on the commitment of an organization that helps to maintain momentum.

The domain of capable consumers focuses on the consumer themselves and their initiative to continue to learn and grow. In this context, the peer support network that was established by PC4 and PoCoG to enable members to support each other was frequently referenced. This programme provided an on‐going mechanism to ensure consumers do not feel isolated, and facilitates long‐term involvement within the team.^22^ Consumers suggested they felt supported and were mentored by other consumers when they needed help which made them feel confident about the feedback they provided to researchers when developing new research projects.

Our results also emphasize that as an organization, greater support of learning opportunities for consumers is important and that consumers’ educational needs do not dissipate over time. Educational support increases consumers’ confidence over time,^7^ making them feel more confident and capable. This organizational dimension has previously been reported as an important contributor to success in consumer involvement.^26^ Continuing training and educational opportunities may also provide consumers with a greater source of credibility in linking their lived experiences and their input objectively.^3^, ^27^ Despite this, some consumers still felt that specific project topics were beyond their scope of experience and did not always feel equipped to review research in a meaningful way. We discovered that although learning opportunities were a facilitator to involvement with consumers, we further identified that some consumers felt it was lacking in specific areas and should be addressed.

The consumer workload was raised as a barrier to long‐term involvement. Consumers discussed the difficulty at times of balancing their consumer role with their personal life and existing commitments and further supported as the main reason members resigned from their role. We know that a substantial proportion of consumer involvement occurs in the early stages of research and study development, with limited input at later stages of the research cycle.^3^ This prominent theme of workload and balance highlights the importance of considering how to best work with consumers throughout the research cycle long‐term and to plan which resources need to be in place to support a balanced workload, in order to ensure enough time for meaningful involvement.^28^ Organizations may benefit by considering the flow on effect of both multiple concurrent funding applications and truncated feedback timelines due to pressure on researchers.^7^, ^29^ These factors may translate to increased burden for consumers and act as a barrier to continued involvement. These barriers suggest that regular review and forward planning are important to proactively mitigate workload burden for consumers.

A lack of diversity in membership, mapped to cultural involvement, was also highlighted by consumers as a threat to inclusivity in the group. In this context, diversity extends not only to cultural background but also gender, age, location, that is rural representation. This lack of diversity and cultural involvement is supported by Hoffman who found that consumer involvement in cancer research is over represented by well‐educated female representatives from ethnic majority groups.^3^ An established barrier is the persistence of insufficient opportunities for vulnerable or minority groups to provide input into research and alternative strategies are needed to successfully reach these groups.^3^, ^4^, ^30^ Though this is not a barrier to individual long‐term consumer involvement, it does speak to the importance from an organizational perspective of creating consumer representation across diverse populations. This may take pressure off consumers to provide input where they do not feel confident and feeds back into the domain of capable consumers and the need to provide educational opportunities. A lack of diversity also potentially limits the ability of the CAG to make meaningful contributions to research strategy development on a broader scale if it does not sufficiently represent the broader community. Moving forward, exploring both potential mechanisms and the impact of creating a more diverse and representative CAG in relation to workload, mentoring and opportunities for peer education and sharing different lived experiences may be beneficial.

The Cancer Australia National Framework for Consumer Involvement in Cancer Control outlines how consumers develop skills over time. So, it is important to retain these consumers whose wealth of lived experience and developed understanding of research can be used to improve the quality of research design. Long‐term consumer involvement may not only be important for harnessing the knowledge of progressively more experienced consumers but also to provide continuity, sustained capacity, minimize on‐going practical and time constraints, and help reduce resource allocation where resources are limited.^3^, ^12^, ^29^

## 6 CONCLUSION

There continues to be a small but growing body of work in relation to effectively involving consumers in health and medical research, including cancer research. This evidence has most commonly focused on either initiating consumer involvement or consumer involvement at different stages of the research cycle.^3^ Additionally, these studies frequently do not provide recommendations on the consumer involvement process.^3^ There is scarce evidence about how to maintain involvement over time and what factors contribute to continuing involvement despite competing pressures. In this context, this study suggests that being an organization that demonstrates commitment to consumer involvement by providing education, training, visible champions and cultivating an environment of mutual respect and equality are key facilitators to involving consumers long‐term. Conversely, a lack of alignment in values, vision, workload and poor communication limit continued consumer involvement in the development of cancer research.

Identifying both individual and organizational barriers to long‐term involvement may help provide a road map for organizations. This road map would be designed to proactively develop solutions to prevent the loss of experienced consumers whose input may significantly improve the quality of new cancer research.

## 7 PATIENT AND PUBLIC CONTRIBUTION

CAG members were the participants of this study. The results of this study and manuscript were reviewed by three CAG member who were not participants.

## CONFLICT OF INTEREST

The authors have no conflict of interest.

## AUTHORS’ CONTRIBUTIONS

K.M conceived the presented research, EA was the consumer representative within the project team, K.M, S.C, J.M and JE undertook data analysis interpretation, K.M drafted the manuscript, and all authors critically reviewed the manuscript.

## Data Availability

The data that support the findings of this study are available from the corresponding author upon reasonable request.
